# Video Head Impulse Test in Children—A Systematic Review of Literature

**DOI:** 10.3390/jcm14020369

**Published:** 2025-01-09

**Authors:** Soumit Dasgupta, Aditya Lal Mukherjee, Rosa Crunkhorn, Safaa Dawabah, Nesibe Gul Aslier, Sudhira Ratnayake, Leonardo Manzari

**Affiliations:** 1 Alder Hey Children’s Hospital NHS Foundation Trust, Liverpool L14 5AB, UK; 2 School of Medicine, Imperial College, London SW7 2AZ, UK; 3 Guys and St Thomas’ NHS Foundation Trust, London SE1 7EH, UK; 4 Otorhinolaryngology, İstanbul Atlas University, Private Doruk Nilufer Hospital, 16110 Bursa, Turkey; 5 MSA ENT Academy Centre, Via T. Piano, 16, 03043 Cassino, FR, Italy

**Keywords:** video head impulse test vHIT, head impulse test, children, paediatric, normative data, VOR gain, review

## Abstract

**Background and Objectives**: The video head impulse test is a landmark in vestibular diagnostic methods to assess the high-frequency semicircular canal system. This test is well established in the adult population with immense research since its discovery. The usefulness and feasibility of the test in children is not very well defined, as research has been limited. This systematic review investigated and analysed the existing evidence regarding the test. The objectives were to derive meaningful inferences in terms of the feasibility, implementation, and normative vestibulo-ocular reflex (VOR gain) in normal children and in children with vestibular hypofunction. **Methods**: Research repositories were searched with keywords, along with inclusion and exclusion criteria, to select publications that investigated the vHIT in both a normative population of children as well as in pathological cohorts. The average normal VOR gain was then calculated in all semicircular canals for both the normal and the vestibular hypofunction groups. For the case–control studies, a meta-analysis was performed to assess the heterogeneity and pooled effect sizes. **Results and Discussion**: The review analysed 26 articles that included six case–control studies fulfilling the study selection criteria, out of more than 6000 articles that have been published on the vHIT. The described technique suggested 10–15 head impulses at 100–200°/s head velocity and 10–20° displacement fixating on a wall target 1 to 1.5 m away. The average VOR gain in the lateral semicircular canals combining all studies was 0.96 +/− 0.07; in anterior semicircular canals, it was 0.89 +/− 0.13, and for posterior semicircular canals, it was 0.9 +/− 0.12. The normal VOR gains measured with individual equipment (ICS Impulse, EyeSeeCam and Synapsys) in the lateral semicircular canals were largely similar (*p* > 0.05 when ICS Impulse and EyeSeeCam were compared). The pooled effect size in the control group was 1, and the heterogeneity was high. It was also observed that implementing the test is different from that in adults and requires considerable practice with children, factoring in the issue of peripheral and central vestibular maturation. Special considerations were suggested in terms of the pupillary calibration, goggle fitting, and slippage and play techniques. **Conclusions**: The vHIT as a diagnostic test is possible in children with important caveats, practice, and knowledge regarding a developing vestibular system. It yields significantly meaningful inferences about high-frequency semicircular canal function in children. Adult norms should not be extrapolated in children, as the VOR gain is different in children.

## 1. Introduction

In the early part of the 19th century, Jean Marie Flourens first identified the role of the semicircular canals in maintaining balance, followed by the discovery and refinement of the vestibulo-ocular reflex (VOR) generated by these canals and effected through the brain and the oculomotor system by Goltz, Breuer, Mach, and Hogyes during the same century [1]. The key function of the VOR is to stabilise the gaze, a key attribute for the sustenance of life and the orientation of oneself in space. Crum Brown was the first to point out the push–pull mechanism of reciprocal stimulation/inhibition of the lateral semicircular canal on each side, the anterior canal on the right with the posterior canal on the left, and the anterior canal on the left with the posterior canal on the right [2]. These groundbreaking discoveries paved the way for future researchers to study the vestibulo-ocular reflex in detail, spearheaded by Barany. However, it was not until 1988, that a clinical test was devised by Halmagyi and Curthoys that could assess the high-frequency response of the semicircular canals by the eponymous head thrust test [3].

The basic principle of the test entails a sharp and fast, abrupt, and unpredictable head thrust with high acceleration and low amplitude on each side of the head at about 10 0rees displacement, whilst asking the subject to focus the eyes on a fixed target. The VOR ensures that the eyes move with the head in the opposite direction to maintain the gaze on the target. This is achieved in different horizontal and vertical planes in the high-frequency response of the canals and is a sensitive and specific indicator of high-frequency canal function [4]. The clinical head impulse test was subjective, contingent on examiner observation and expertise; therefore, there was a need for objective quantification. In 1988, Cremer first used a scleral coil to physically measure the VOR in response to a head thrust [5]. In 2005, Ulmer and Chay devised a remote mounted camera to capture and quantify the eye movement [6]. Further refinement with scleral coil experiments that are applied close to the pupil led to the discovery of the video head impulse test (vHIT) by MacDougal et al. [7] in 2009, where an infrared camera is applied close to the pupil through a head band for lateral semicircular canals that was found to correlate well with the scleral coil measurements. The same team in 2013 [8] then devised a technique for measuring paired vertical canals’ VOR as well.

In the normal situation, the VOR ensures that the eyes remain fixed on the target, and the ratio of the head movement to the slow phase eye movement generated by the VOR is defined as a VOR gain that approaches unity and is equal. In deficiencies of the VOR, the eyes move with the head in the same direction as the head movement. The brain recognises this error in response to the command and generates a catch-up quick movement, called a saccade, to bring the eyes back to the target. Since the eyes cannot move in the direction of the VOR, the VOR gain involving the slow phase of the VOR becomes far less than unity, and the saccade ensures that the VOR is still maintained. The slow phase of the VOR gain and the saccade are clearly discernible in the vHIT measurement device. The difference between the clinical and the software-driven vHIT are two-fold—firstly, in the former, one cannot measure the slow phase of the VOR gain, and secondly, in the former only an overt saccade, i.e., the saccade that is generated after completion of the head movement can be seen; however, a covert saccade, i.e., the saccade generated during the head movement, cannot be seen. In the latter, both can be seen, and a VOR gain can be measured.

Since its discovery, the vHIT has seen intense research and is now established as a key investigation to quantify high-frequency canal function in all canals and on both sides individually. This is a milestone in vestibular diagnostics yielding valuable information crucial for diagnosis and management. Furthermore, it has also been identified as providing valuable information for vestibular compensation by virtue of the analysis of the saccade morphology [9]. vHIT features in the Barany diagnostic criteria for bilateral vestibular hypofunction [10] and in the proposed isolated otolith disorder criteria by Park et al. [11].

Whilst the vHIT has been studied extensively in adults, it has rarely been studied in children. Vestibular disorders in children generate a significant morbidity affecting not only balance but overall development, including cognitive development in children [12]. The prevalence was estimated to be between 0.45 to 5.3 in a U.S. study in a nationally weighted population ranging from 278,000 to 3,278,000 [13]. Thus, accurate diagnosis is essential, including quantification, as this leads to rewarding management outcomes. To this end, the vHIT, as a diagnostic marker, plays an important role.

The fundamental difference between adults and children as far as VOR is concerned is that the VOR in children is in the process of development and maturation, and thus, it would be incorrect to transpose adult norms to children. The standardization of vHIT norms require scrutiny and analysis because these can be quite heterogenous across different centres dealing with different paediatric population cohorts. A paediatric head impulse test requires far more practice and special paediatric training than in adults, with expertise that might not be available. Only a few centres in the world assess children with vHIT. Therefore, there is a gap of knowledge about the test in the paediatric population in terms of the efficacy, ease, and interpretation.

This article provides a systematic review of the vHIT in children, which has not been attempted before. The aims are to study the feasibility and the ease of the test in the paediatric population and to quantify aggregated norms of the different parameters of the vHIT in children, as gleaned from published evidence in the normal population. The objectives are to draw meaningful inferences about the outcomes of the test by calculating a pooled average VOR gain in the normal population of children for reference for future studies. A secondary objective was a scoping review of the VOR gain in vestibular pathologies in children and an estimation of the pooled effect sizes in case–control studies involving cohorts of normal children and children with vestibular disorders.

## 2. Methods

### 2.1. Search Strategy

We conducted an integrative literature search of the published evidence pertaining to the video head impulse test in children. It was designed based on the guidelines of the Preferred Reporting Items for Systematic Reviews (PRISMA). The keywords used were video head impulse test vHIT, head impulse test, children, paediatric, normative data, VOR gain, and review. The repositories and databases searched included PubMed, Scopus, Google Scholar, Cochrane reviews, Science Direct, Embase, and Ovid. The review was not registered with Prospero, a systematic review database, as the data collection was complete before registration. All data from the review are included in the main text.

### 2.2. Review Questions

What are the reference values of the VOR in normal children across all semicircular canals? How should one perform the vHIT test in children, and how easy or difficult is it? What is the VOR gain in vestibular pathologies in children, and how does it differ from normal values?

### 2.3. Inclusion Criteria

The inclusion criteria included all studies reporting a normative value of the vHIT in children from 6 months to 18 years. Studies reporting a pathological cohort with or without a normal population were included. Studies that reported useful information regarding the test procedure in children were also included for discussion.

### 2.4. Exclusion Criteria

Specific pathological cohorts (for example vHIT in children with sensorineural hearing loss, cochlear implants, specific genetic disorders), abstracts, series with less than 10 subjects, review articles, and case reports were excluded.

### 2.5. Data Extraction and Analysis

First, all studies with the designated key words were obtained. The studies were then subjected to an initial screening for the elimination of studies in adults by all authors. This was followed by the elimination of studies that reported vHIT in specific vestibular disorders in children, as well as studies that were case reports or a case series and abstracts by all authors. The selection thereof was then analysed by 3 senior authors who are paediatric vestibular specialists and who have performed thousands of paediatric vHITs among themselves and published their own laboratory norms in a tertiary paediatric vestibular unit. Agreement or concordance with the study question was then analysed. The final list was further analysed by obtaining the mean VOR gains across the canals. Since the reporting was variable, especially because different age cohorts were reported and sides considered separately in some studies, an average VOR gain was considered for the right and the left sides from studies that reported the gains separately for each ear. The scientific rationale for this pooling of VOR gains across different age groups and sides lay in the observation that they were not significantly different from each other, which would be clinically relevant. The VOR gain values as a function of age are consistent over a wide age range from 20 to 80 years with a slight decrease in old age [14,15]; however, the issue in the paediatric cohort is less well determined, and this review was envisaged to provide some insights. We also studied the individual and combined cumulative VOR gain values of all 3 types of equipment used in different studies, namely the ICS Impulse (Denmark), the EyeSeeCam (Denmark), and the Synapsys (France) system.

The studies that reported vHIT in a pathological cohort only were analysed for meaningful inferences, and a metanalytic quantification was not possible, as normative gain values were not given, and the number of studies was small. The case–control studies were separately analysed for metanalysis of pooled effect sizes in terms of lateral semicircular canal gains that also yielded an inference of heterogeneity in these studies. Vertical canal gains in these studies were not available in all instances thereby precluding a metanalysis of vertical canals.

One study reported the effect of the peak head velocity on VOR gains that was analysed separately and not included in the metanalysis. Some studies compared the vHIT with the caloric test and the rotatory chair test to assess the correlation and agreement and formulate the sensitivity and specificity of the vHIT. These were not analysed separately whilst reviewing these studies, as the vHIT is a fundamentally different test from either the calorics or the chair, as the former assesses the high-frequency function of the vestibular sensory epithelia, and the latter ones assess low-frequency responses.

### 2.6. Quality Analysis

The scientific quality of the publications in terms of content and answering the research question was evaluated by a senior author and cross checked by another senior author in the studies that underwent quantitative synthesis using the mixed methods appraisal tool (MMAT) version 2018 [16] (*n* = 19). Quantitative descriptive methods were assessed, and an overall rating from poor to reasonable and good was applied.

### 2.7. Statistical Methods

The averaged VOR gain across the studies was analysed using Social Statistics, an online statistical calculator (https://www.socscistatistics.com/tests/—accessed 2 December 2024). A paired *t* test with a confidence interval of 0.05 was performed to compare the VOR normal gains between the ICS Impulse and the EyeSeeCam systems. A second statistical software developed by Stats Direct Ltd. and The University of Liverpool supported by Leap of Faith Ventures https://www.statsdirect.co.uk/Default.aspx (downloaded 2 December 2024) was used for the metanalysis of the effect sizes across the case–control studies. For the studies that reported side-specific lateral semicircular VOR gains, pooled/average standard deviations (SD) were calculated by the formula for averaging standard deviations across a same sample size by the formula √s1^2^/2 + s2^2^/2, where s1 is the SD of the left lateral semicircular canal and s2 the SD of the right semicircular canal.

**Figure 1:**
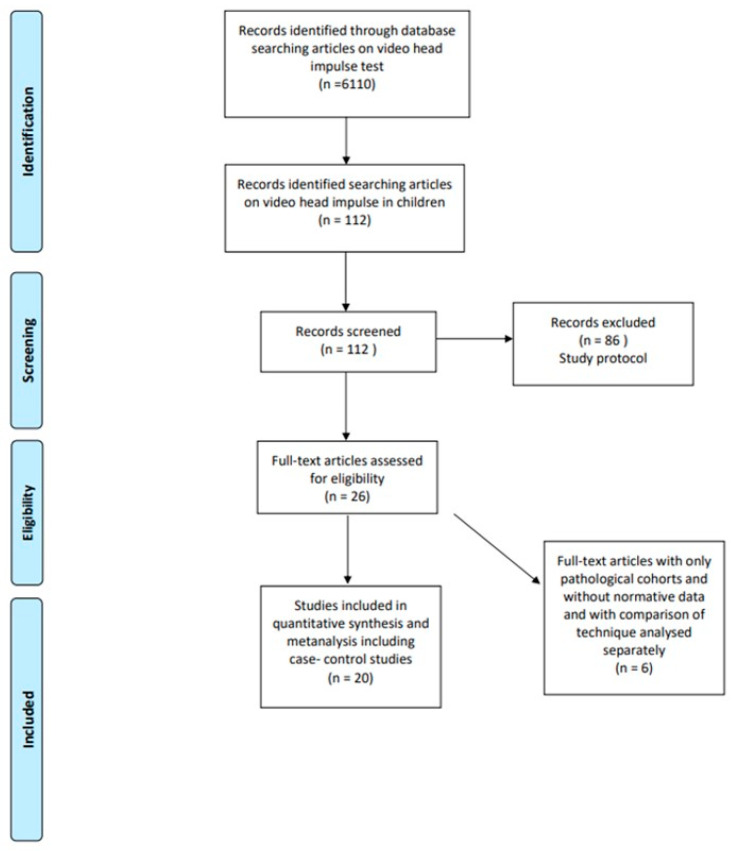
PRISMA flow chart.

The PRISMA algorithm is given in Figure 1.

## 3. Results

### 3.1. Article Output

Using the designated key words in research repositories, a total of 6140 articles pertaining to video head impulse test published in English language only were observed from 2009, the year of first publication, to the present day. This was then further streamlined or fine-tuned with the words children and paediatric that narrowed down the search to 112. These articles were then screened by the study protocol, and 86 studies were excluded. Of the remaining 26 articles [17,18,19,20,21,22,23,24,25,26,27,28,29,30,31,32,33,34,35,36,37,38,39,40,41,42], five studies [23,25,29,30,38] investigated pathological cohorts only without any data on normative values. One study [41] investigated the relationship between the peak head velocity and VOR gain in children. Institutional unpublished data from a published audit and a personal series were included.

The final list of studies that reported values in normal and mixed normal–pathological cohorts and assessed for quantitative synthesis, therefore, included 20 [17,18,19,20,21,22,24,26,27,28,31,32,33,34,35,36,37,39,40,42]. Of these, six [24,27,28,34,40,42] were case–control studies investigating both a normal and a pathological group. Fourteen [17,18,19,20,21,22,26,31,32,33,35,36,37,39] studied a normal population only.

### 3.2. Demographics of Children Studied and Study Characteristics

A total of 1691 children between the ages of 5 months and 18 years were studied in the 25 studies reporting vHIT results in normal and in pathological cohorts. The normal cohorts consisted of 1189 children, and the rest comprised pathological cohorts (502). Twenty-one studies were prospective except four [23,25,29,38] that were retrospective studies. Seven studies [21,27,29,31,33,35,38] investigated only the lateral semicircular canals whilst the remaining fourteen investigated all canals. Ten studies did not report gender [17,20,23,30,31,35,37,38,39,42].

Only three studies studied vHIT in children less than 3 years of age [23,33,39]. Six studies used the EyeSeeCam system [21,27,31,32,35,36]. Fourteen used the ICS Impulse system [17,18,20,22,24,25,26,28,29,30,37,38,40,42], and four studies used the Synapsys system [19,23,33,39]. One did not mention the equipment [34]. The three studies that studied vHIT under the age of 3 years utilised the Synapsys system. Some studies using the ICS Impulse system outlined the non-availability of a suitable size headband to fit a child under the age of 3 years, using foam pads for a good fit and the importance of tightly fitting goggles for meaningful inferences in the test [18,20,22,24,25,26,29,35,36,40]. The studies using the EyeSeeCam system alluded to the problem of goggle slippage in a mobile camera [32]. Under the age of 3 years, the tests were performed with the child sitting on the carer’s knees.

Nine studies [20,21,31,33,35,36,37,39,42] reported VOR gain across different age groups and did not observe any statistically significant differences in the VOR gains across the paediatric age group from the age of 6 years. The Martens study [33] observed that children under the age of 1 year showed statistically significantly lower VOR gains than other groups, whilst the Rodrigues Villalba study [36] showed that children from 3 to 6 years old showed statistically significantly lower gain values than older children. Wiener-Vacher pointed out that refractive errors under the age of 3 years contribute to this gain value [39].

**Figure 2:**
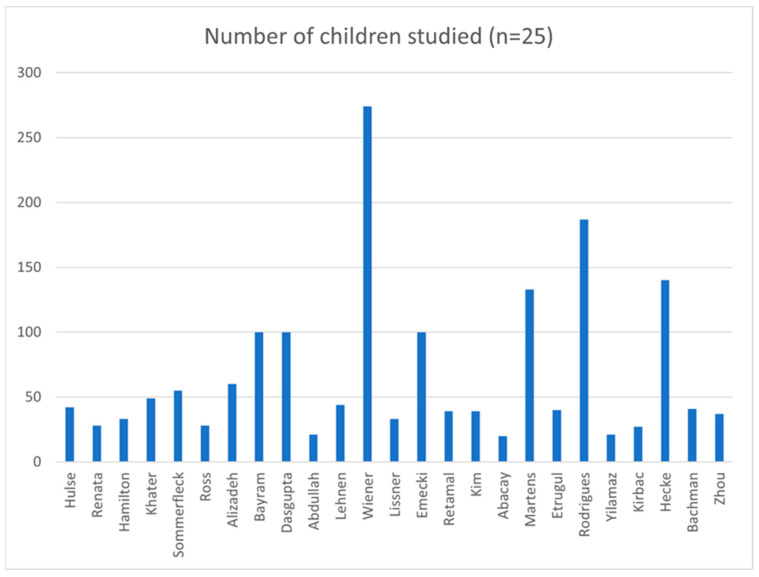
Number of children studied, represented on *y* axis.

**Figure 3:**
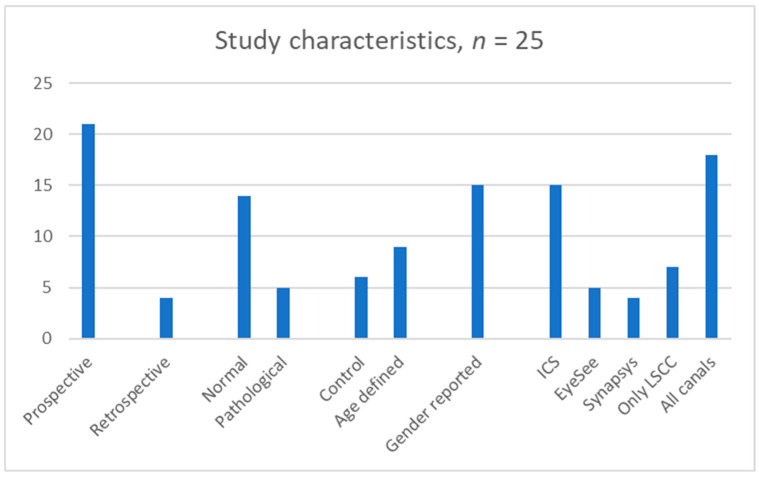
Study characteristics: ICS—ICS Impulse; EyeSee—EyeSeeCam; LSCC—Lateral semicircular canal; number of studies is represented on *y* axis.

Figure 2 and Figure 3 illustrate the demographics and study characteristics.

### 3.3. Technique and Feasibility

Twelve studies reported the time required to perform the test in children, which was observed to be quick [18,20,24,26,27,28,30,31,37,38,39]. Seven studies calculating the time reported an average time of 10–15 min [24,26,27,30,31,37]. The technique involved unpredictable head impulses delivered on an average for at least 10 and up to 20 impulses [21,22,24,25,26,27,28,29,30,32,34,35].

The distance between the target and the pupils on an average was 1 m with a fixed target on the wall, which was recommended to be an attractive target, for example toys that would appeal to the children, in almost all studies. Many studies reported on the importance of adequate instructions and tailoring the test according to the child’s needs for example avoiding blinking and adequate engagement [18,24,25,26,27,29,32,34,35,39], and a further four studies recommended a technique whereby the eyelids were recommended to be pulled up [18,20,24,26].

The displacement of the head was reported by most studies as 10–20° whilst executing the impulses at a peak head velocity between 50 and 250°/s with an average of at least 100–150°/s. Whilst high velocities were possible in lateral head impulses, as many as five studies [23,33,36,37,39] reported that a similar high peak velocity was difficult to achieve in the vertical canals.

A couple of studies [29,40] specifically mentioned operator variability that could generate heterogenous results. All studies reported on the ease of performing the test with practice and experience. The test–retest variability was minimal, as reported in one study [37]. One study [21] observed several reasons for possible test failure that included apprehension, lack of interest, and failure of calibration.

Table 1 illustrates the different studies and important observations, whilst Table 2 is a representation of VOR gains and the demographics.

### 3.4. VOR Gain Across Studies

The mean normative value of the VOR gain reported across 20 studies that reported normative data in the normal population [17,18,19,20,21,22,24,26,27,28,31,32,33,34,35,36,37,39,40,42] was evaluated statistically. The average VOR gain was observed to be 0.96 +/− 0.07 in the lateral canals, 0.89 +/− 0.13 in the anterior canals, and 0.9 +/− 0.12 in the posterior canals. Whilst there was large concordance within the lateral semicircular canal gain, there was variability noted for the vertical canal groups especially the anterior semicircular canal, where at least three studies reported gain values of lower than 0.8 [24,26,42].

The mean VOR gain for lateral semicircular canals in the normal groups was 0.98 with the ICS Impulse system (*n* = 10), 0.95 with the EyeSeeCam (*n* = 6), and 0.95 with the Synapsys system (*n* = 3). The EyeSeeCam system reported vertical canal gains only in one study [36], whilst the Synapsys system recorded vertical canal gains in all three studies. The ICS Impulse studies reported an average gain of 0.87 in the anterior semicircular canals and 0.89 in the posterior semicircular canals, whilst the Synapses system recorded an average gain of 0.97 and 0.93 in the same canals, respectively, with variability noted in the anterior semicircular canal VOR gain. We were unable to compare the system outputs statistically as the dataset did not meet the statistical threshold due to only a handful of studies with the Synapsys and the paucity of vertical canal data with the EyeSeeCam. However, the data did permit a paired *t* test between the ICS Impulse and the EyeSeeCam when comparing lateral semicircular canal normal VOR gains. This indicated a *t*-value at 0.81417 with a *p*-value of 0.214589. The result was not significant at *p* < 0.05.

**Figure 4:**
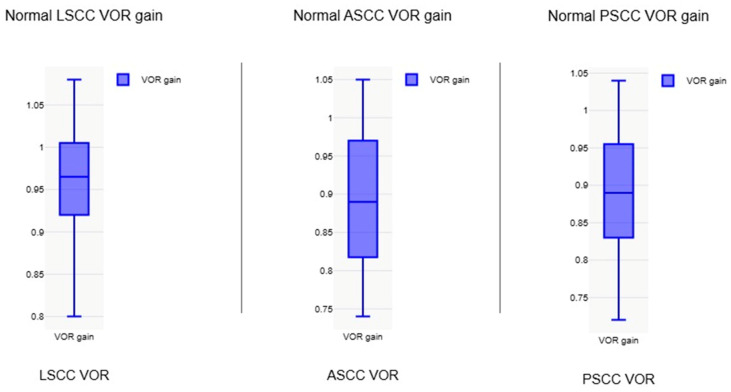
Average normal VOR gain in lateral, anterior, and posterior semicircular canals *n* = 20; VOR—vestibulo-ocular reflex, LSCC—Lateral semicircular canal, ASCC—anterior semicircular canal, PSCC—posterior semicircular canal.

**Figure 5:**
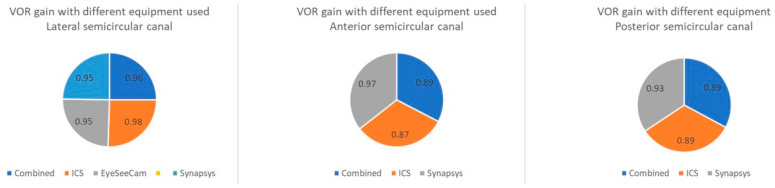
Average normal VOR gain with different equipment used.

Figure 4 presents a box and whisker plot of the average VOR gain in the normal population in different canals. Figure 5 shows the VOR gain with different equipment used for lateral, anterior, and posterior semicircular canals.

When comparing the VOR gain value across the lateral semicircular canal plane in studies which included data for both normal and pathological groups [24,27,28,34,40,42] (*n* = 6), the combined random effects pooled effect size was significant with a large effect size of 1 and a significant heterogeneity (I^2^ inconsistency 86.2%). Two studies [27,29] reported a VOR gain value of less than 0.5 in their pathological cohorts as compared to other studies who reported VOR gain values in their pathological cohorts between 0.6 and 0.8 (average gain across all studies 0.67 +/− 0.16). All these studies reported statistically significant VOR gain differences between their normal and their pathological cohorts. These studies, in addition to the studies that reported a pathological cohort only [23,25,29,30,38], also observed that a low VOR gain with corrective saccades is the strongest indicator of vestibular weakness. VOR gain alone was not a strong indicator of vestibular weakness as commented on by two studies [25,37]. In fact, five studies [24,28,29,31,42] reported that refixation saccades with normal VOR gain indicated compensated vestibular weakness.

**Figure 6:**
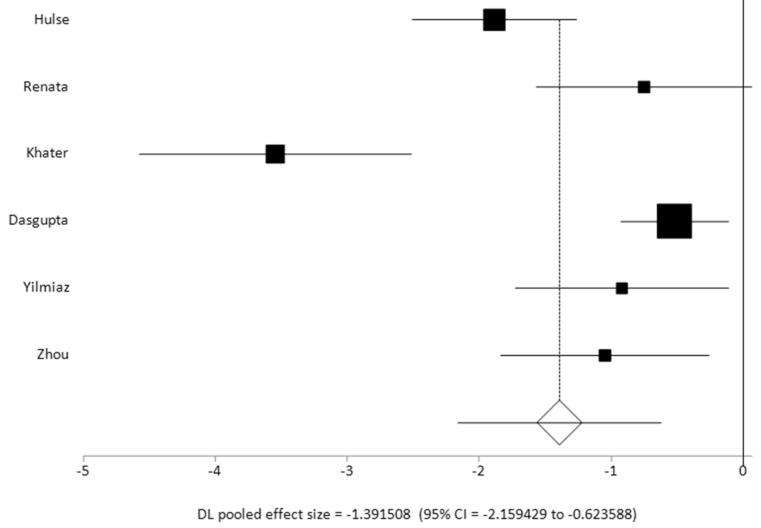
Pooled effect in control studies *n* = 6.

Figure 6 presents a forest plot of the pooled effect sizes of controlled studies that were meta-analysed.

The five studies that studied the vHIT only in a pathological cohort [23,25,29,30,38] reported individual gains in the specific aetiologies that they encountered. A VOR gain value of less than 0.7–0.8 with refixation saccades was deemed abnormal. Hamilton [25] reported that saccades were 100% specific and sensitive to confirm vestibular weakness. Sommerfleck [38] considered balance disorders in children as a whole and observed that about 42% presented with vestibular symptoms. Many of these children showed a diminution in the VOR gain in vestibular pathology except in vestibular migraine. Kim [29] pointed to several sources of artifact contamination: specifically blink artifact, difficulty with following instructions, inattention, and goggle slippage. They reported a low sensitivity but a high specificity when compared to the caloric test. Etrugul [23] observed saccade morphology evolution with vestibular compensation and, whist calculating average gain across all canals, described a significantly low gain of less than 0.7 in vestibular pathologies. Kirbac [30] observed similarly, reporting significantly low gains across a pathological group.

There was only one study that investigated the peak head velocity with VOR gain [41]. This demonstrated that achieving high velocities in the vertical canals was more difficult than in the lateral canals, but since the VOR gain was not different with different peak head velocities, including slower, it was felt to not have contaminated the outcome. Wiener came to the same conclusion [39].

Table 3 summarises the statistical analysis.

### 3.5. Quality Analysis

**Figure 7:**
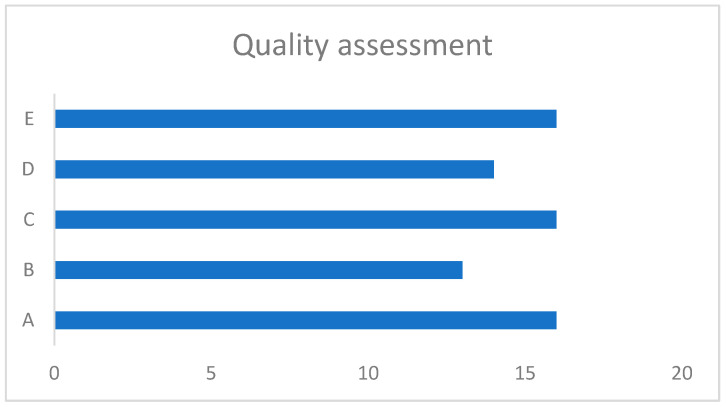
Quality analysis (*n* = 19). Legend: A: Addressal of research question; B: representativeness of target population; C: appropriate measurements and statistical methods; D: low nonresponsive bias; E: good/reasonable quality.

Clear research questions were described in all but two studies [34,40]. The sample strategy relevant to address the research question was deemed satisfactory in 16 and undetermined in 3 [27,34,40]. The samples were deemed representative of the target population in 13 studies [17,20,22,25,26,28,29,32,33,35,36,38,42]. Sixteen studies included appropriate measurements and statistical analysis [17,18,20,21,22,25,26,28,30,31,32,33,35,36,39,42]. Fourteen studies were considered to have low nonresponsive bias [17,18,20,22,25,26,28,30,31,32,33,36,39,42]. Eleven studies were deemed to be good, five were reasonable, and three were poor, as shown in Figure 7.

### 3.6. Recommendations

We provide recommendations as to the different aspects of the vHIT in children in Table 4 as gleaned from this review, based on Table 1 and Table 2.

## 4. Discussion

The video head impulse test has revolutionised vestibular diagnostics yielding valuable information about high-frequency vestibular canal function. This frequency is the most utilised of all frequency responses in the human species and, therefore, the most practical one in day-to-day function. Previously the calorics and rotatory chair yielded ear specific information for low-frequency vestibular lateral canal function only. The vHIT is not only side-specific but yields information on all semicircular canals.

Whilst the efficacy, significance, and feasibility of this quick non-invasive test is well-established, evidenced by over 6000 publications over the last 15 years, there were only 112 studies that had studied the test in children. Out of these, a vast majority researched the vHIT in specific pathological cohorts, for example in sensorineural hearing loss or cochlear implantation children or individual disorders known to cause a hearing loss. These studies did not include normative data or a comparison of the test outcomes with other pathologies, especially the ones that spare the cochlea. Therefore, these studies were excluded. Along with other exclusion criteria, our final list of studies who reported normative data and cumulative pathological data comprised 25 articles. All these studies also commented on the test logistics and feasibility in the paediatric population. Consensus is yet to be achieved as to the technique of the test in children that provides most informed outcome, and we felt that by looking into the evidence, we could determine one.

Our main research objective was to quantify normal VOR gains in the paediatric population. As can be noted, our review articles were quite heterogenous with different study designs and protocols, and the normal VOR gains were likely to differ. However, there was good consistency in reporting lateral canal VOR gains across the study groups, with an average value of 0.96 +/− 0.07 (range 0.8–1.08) in the lateral canals. This would translate to a VOR gain between 0.9 and 1 as a normal value for VOR gain for this canal in children across age groups, similar to that in an adult. This gain value was less than 0.9 in the Martens study [33] under the age of 1 year, which was statistically lower than older children, but it can be noted that this lower gain value is still more than 0.7, which was apparent in pathological cohorts in this review. Thus, a gain value between 0.7 and 0.9 in children under the age of 1 year does not appear to be clinically significant unless accompanied by other stigmata and signs of vestibular disorders in children.

When the anterior semicircular canal VOR gain was pooled, it was noted that this was 0.89 +/− 0.13 with a range of 0.74 to 1.03 as the normal values. For the posterior semicircular gains, the pooled average was 0.9 +/− 0.12 with a range between 0.72 and 1.04. This is lower than that found in adults, which is 0.8 and above. In fact, four studies [18,24,36,42] observed gains of less than 0.8 and just over 0.8 in the anterior semicircular canal and in the posterior semicircular canals, respectively. A low normal VOR in the vertical canals gain is attributed partly to the cervical neck maturation and cervical neck muscle tone in a developing child according to three studies [21,24,37]. The vertical canal gains showed greater variability, breadth, and outliers than the lateral canal gains as can be seen in the figures.

The VOR gain in pathological cohorts with vestibular problems was reported in studies that determined this to be uniformly lower than the normal value (<0.7) and combined with refixation saccades, the most important indicator of vestibular weakness that is well-known (five studies with pathological cohorts and six studies with case–control cohorts). When we compared the case–control studies for the combined effect size of VOR gain in the lateral semicircular canals, we detected a large effect size that is not unsurprising and well-accepted in the adult population. We also observed significant heterogeneity in this group of studies when they reported the VOR gain in the pathological cohort in the lateral semicircular canals. This was because the Hulse [27] and Khater [28] studies reported a significantly lower gain of <0.5 as compared to the others ranging between 0.6 and 0.8 in their pathological cohorts. This can be explained by the observation that the pathologies in the cohorts in each study were different. Furthermore, since the VOR gain improves with vestibular compensation, especially in a highly plastic and maturing vestibular system in the paediatric population, the data captured in a given time frame will be variable as well. However, the mean VOR gain showed an average of 0.68, which is well below the normal range, as described earlier.

Therefore, it can be said that a high-frequency VOR as measured by the vHIT is as effective a biomarker of vestibular weakness in children as in adults, notwithstanding a maturing and developing vestibular system in children. The VOR is present at birth but is not mature and reaches functional maturity around 6–12 months of age [39]. As it matures, it calibrates and refines according to environmental stimuli. This also underpins the very important role of the developing VOR in children from an early age. We believe that this observation suggests that derangement of the VOR in children due to any reason will affect a child’s orientation in space and balance that can be eminently detected by the vHIT; therefore, this test needs to be performed to investigate balance disorders in children.

Five studies reported normal gain with refixation saccades as a further indicator of compensated vestibular weakness [24,28,29,31,42]. This observation coupled with the observation that VOR gain alone was not the sole marker for a vestibular weakness and the high sensitivity and specificity of saccades noted in one study lead one to infer that saccades are crucial to establish vestibular weakness. In adults, studies by Perez Fernandez [43] and replicated by Korsager [44] also demonstrated that refixation saccades with normal VOR gain suggested compensated vestibular weakness. It must be borne in mind that vestibular compensation in a child will be different from those in adults due to the evolving and highly efficient and active cerebral plasticity. Thus, VOR gain morphology and saccade evolution will be different too and, likely, more noticeable. A recent SHIMP study in children arrived at a similar conclusion [45]. It is likely that further qualification of saccade and other related parameters (such as, amplitude and latency) might evolve in children in the future.

The studies used three different types of equipment for the vHIT—ICS Impulse (head-mounted fixed camera in nearly two-thirds of the studies), EyeSeeCam (head-mounted mobile camera), and Synapsys (remote camera). We combined the VOR gains of all studies to formulate the guidelines, although we are aware that the gain values may be significantly different from each other with different equipment. In this review, gains for individual equipment were well within range of the cumulative average gain values (Figure 5), except in the anterior semicircular canal. Furthermore, the normal lateral semicircular canal gains obtained with the ICS Impulse system and the EyeSeeCam were not statistically different from each other. Other statistical comparison could not be conducted among the three systems due to the very small number of studies utilising the EyeSeeCam and the Synapsys systems. We note the heterogeneity in vertical canal gains in our review that might be explained by the use of different equipment in different cohorts of a heterogeneous paediatric population. Differences in output comparing the three systems have been reported in adults [46]; however, in the same study, it appears that only a few significant differences were observed, but this had no impact on the normality of the gain, which remained within the normal reference values [46]. A further study demonstrated that there was an 83% concordance between the three systems in vestibulopathies in adults. Significant differences appeared when the cut off was 0.6 [47]. No studies exist in children as to whether the gains would be significantly different from each other with different equipment as yet, and until that is available, we consider that they will not differ significantly from each other to the extent that they will be clinically significant as in adults, and as this review observed.

Most studies in this review investigated children over the age of 3 years with the exception of three studies [23,33,39]. These investigators used the remote camera Synapsys system. Head bands fitting small heads under the age of 3 years or slightly over are not yet manufactured or marketed, although individual centres have improvised various techniques. The common technique is to use extra foam or padding in the head band for the ICS Impulse and the EyeSeeCam systems using a head-mounted camera as suggested by two authors [18,20] to avoid goggle slippage. Tight and optimal fitting goggles to obtain meaningful results through the band were recommended by several studies (*n* = 10). This is intuitively a most important consideration in children given the head circumferences, and a band not fitted correctly is a recognised cause of skewed VOR gain [48]. A remote camera system that precludes the use of a head-band mounted camera can be tried from the age of 6 months onwards [33]. One study [32] reported goggle slippage in a mobile camera system that can interfere with VOR gain calculations.

Bayram [21] pointed out several reasons that precluded successful implementation of the test. The current authors agree to this and have encountered the factors mentioned that included fear, anxiety, disinterest, and calibration failure. However, even in these circumstances, these factors can be mitigated by increasing play and engagement with the children to make the test interesting, especially in children with neurodevelopmental conditions and to overcome apprehension. In some instances, calibration is impossible, where default calibration can be used. This is not ideal, as in all software, the default calibration is based on an adult population. Even then, vHIT should be attempted and the results interpreted using a holistic approach to aim to draw meaningful conclusions.

Pupillary calibration was deemed important across several studies (*n* = 7), which was recommended to be attained by lifting the eyelids up and by performing the test in a bright room to avoid pupillary constriction. Technical difficulties for the test practitioner especially in very young children are possible due to the difficulties in following instructions by children and their tendency to be easily distracted.

All studies emphasized the need for correct and adequate instructions whilst performing the test by explaining carefully and repeatedly encouraging the subjects and the carers alike. Attractive targets were suggested to be chosen for gaze fixation, and special care was recommended to engage the children at all times to avoid flagging attention that can make the test difficult to perform. Anxiety has been known to influence VOR gains [49]. The time taken to implement the test ranges from 10 min to 20 min and is deemed to be quick. The lesser the time taken, the more engaged the children are likely to be for a reliable test. This underpins the vital suggestion that was a common theme across all studies that experience and practice are required to perform this test successfully in children. The present authors cannot emphasize this point well enough; paediatric training is pivotal to perform the test. A study by Money-Nolan [14] investigated the likely cause of noise in vHIT measurements and identified operator variability, google tightness, gaze alignment towards the canal plane, and inconsistent technique, which generates unreliable VOR gains, which is also true for the paediatric population as well. They recommend adopting consistent techniques and individual laboratory norms that we agree to.

In terms of technique, the review observed that a minimum of 10 impulses for all canals with about 10–20° displacement at a peak head velocity of 100–200° per second should elicit a meaningful and robust response of the vHIT. It is important to obtain at least 10 trials to reproduce or replicate the output results, as invariably some impulses will contain artifacts, especially in children, due to the reasons enumerated above, for example blink artifacts, lack of attention, and pupillary calibration. The distance of the target should be 1 m. Vergence determined by distance does not play a role according to Wiener-Vacher [39]. Some studies have pointed out that it is difficult to achieve sufficient head velocity for the vertical, especially the anterior, semicircular canals. Low peak head velocities may affect gain as observed in adults. Furthermore, in adults, low peak head velocities may not elicit the high-frequency VOR, as smooth pursuits contribute to gaze stability rather than the VOR [14]. In children, this smooth pursuit system does not develop until late, so low peak head velocities should not contaminate the VOR gain in children [50]. Zhou [41] observed that in children, peak head velocities of different magnitudes did not alter the VOR gain significantly. Therefore, the lower peak head velocities to assess vertical canal function still yield canal-specific VOR gains that are of use diagnostically.

Age did not influence any significant changes in VOR gain in the studies that investigated the vHIT in different age groups from the age of 6 years. The study [33] using the Synapsys remote camera system successfully performed the test in children under 1 year of age, where they found that the VOR gain under 1 year was significantly lower than those at 3 years. The study [36] using an EyeSeeCam system noted that children between 3 and 6 years demonstrated significantly lower gain values than older children. This study did not separately analyse the VOR gain at 3, 4, 5, and 6 years, and thus, it is likely that the low VOR gain was mainly observed at 3 years. This is not unsurprising, as high-frequency VOR function matures until the age of 2 years [39,51]. In addition, hypermetropia and astigmatism in children under the age of 3 years are also responsible for this gain variability [39].

Most studies stressed the importance of supplementing the vHIT with other vestibular function tests like the calorics, the rotatory chair, the VNG, and the otolith vestibular-evoked myogenic potential tests. We consider this recommendation as a sound approach to glean comprehensive information about vestibular function in children. Since the vestibular sensory epithelium response is tonotopic or frequency-specific, there may be conditions where selective frequencies can be affected. Thus, one test assessing a high-frequency response like the vHIT is unlikely to give a complete picture. Furthermore, we recommend that the output from the test in children, as in adults, should be analysed by the clinician followed by peer review if necessary and taken holistically along with the clinical phenotypes and other vestibular tests for most effective and meaningful information.

We have provided recommendations for paediatric vHIT in terms of technique, test parameters, and output, as gleaned from the review. These recommendations are envisaged to serve as a guideline for centres that perform vHIT in children. This should not be considered as a definitive caveat. We emphasize that this review article does not attempt to discuss the nuances, pros, and cons of the test itself, as this is evolving and is frequently discussed in the adult literature. We have reviewed the existing literature in the paediatric population from robust studies, in which researchers performed the test with due consideration to the artifacts and logistics that can be encountered, the calculation of the VOR gain, and the practice, experience, skills, and knowledge required to execute and interpret the test in children. We are mindful that adult norms cannot be simply extrapolated to children, and a paediatric vestibular physician must be well-versed to interpret the test results in the lights of a clinical phenotype, supplementary tests, and overall development in children. We are aware that although the vHIT is a good test to quantify high-frequency canal function, it is not a simple ‘put it on and do it’ device, and there are variables that can generate spurious values. The papers selected for this review considered these variables and, therefore, can be considered for recommendations. Our objective was to study the test as reported in the paediatric population and attempt to standardise the test for clinical purposes as a guide. We affirm our stance that every individual paediatric department must obtain their own norms with each type of equipment they use.

This review was limited firstly by the low number of studies that have investigated vHIT in the paediatric population. Secondly, the gold standard of prospective randomised controlled trials is lacking in the subject. Thirdly, given the limited availability of data, a full meta-analysis was not possible. Fourthly, we combined studies using all three different systems to assess VOR gain. Studies comparing the VOR gain with these three types of equipment do not exist in the paediatric population, and moreover, unlike in adults, paediatric norms have not been published by the manufacturers. Therefore, it is difficult to understand how these three systems differ from each other in the same group of children, which can be considered a limitation. Fifthly, there is no gold standard test to validate vHIT in children to compare high-frequency canal function, as scleral coil testing is not appropriate in children. Hence, diagnostic accuracy will be difficult to ascertain, reiterating the previous point that other tests are recommended to be performed for a full diagnostic vestibular work up in children. Sixthly, vHIT is not a treatment; hence, it cannot be compared to a sham procedure. The VOR gain value on its own should not lead to a meaningful inference about hypofunction, and consideration of refixation saccades are rather important to infer weakness.

However, the strengths of the studies are apparent. The quality assessment indicated that more than 80% of studies yielded meaningful conclusions. The majority of them were prospective with adequate statistical methods applied to a representative population of children including six controlled studies that eliminated the bias of retrospective analysis. The majority utilised one system to perform the test, i.e., ICS Impulse (>50%). Most studies (80%) investigated a normal population either as the only objective or when compared to a pathological group. Therefore, this review suggested a robust average VOR gain value in normal children, a cut off for vestibular weakness, and the importance of refixation saccades in making meaningful inferences about vestibular pathology in children.

## 5. Conclusions

We feel that this review will set standards for performing the vHIT in children and benchmark parameters due to its strengths. Nevertheless, we also emphasize that individual laboratories should ideally establish their own norms in children for consistency. The procedure requires adequate paediatric experience and practice to eliminate artifacts. vHIT is a highly feasible, non-invasive, and an eminently doable investigation that can be performed in children. Crucially, this review has highlighted the special considerations in terms of the actual test and its outcome that differs from adults, reiterating a most important caveat that adult testing paradigms cannot be extrapolated directly to children.

## Figures and Tables

**Table 1:** Studies and salient observations in studies *n* = 25.

Study	Equipment	Method	Time Taken	Comments
**Hulse 2015 [27]**	EyeSeeCam Lateral canal only	At least 10 impulses with 5–15° lateral displacement at 100–200°/s 1 m target	20 min	1. Under 6 difficult to follow instructions 2. Use of attractive targets 3. 76% reproducible results—cervical neck muscles preclude impulses 4. Instructions are important 5. Consistent with pathology
**Renata 2015 [34]**	Not given All canals	20 impulses with 20° displacement at 70–170°/s 1 m target	Fast	1. Instructions are important 2. Brightly lit room
**Hamilton 2015 [25]**	ICS Impulse All canals	Not given	Not given	1. Corrective saccades more 100% sensitivity and specificity 2. VOR gain 66% sensitivity and 100% specificity 3. VOR gain < 0.7 pathological and consistent with pathology 4. Blink artifacts 5. Goggles cannot be fitted under 3 years
**Khater 2016 [28]**	ICS Impulse All canals	20 impulses with 5–20° displacement at 50–250°/s 1 m target	Quick	1. Corrective saccades may exist with normal VOR gain in pathology 2. High sensitivity and specificity
**Sommerfleck 2016 [38]**	ICS Impulse Lateral canal only	15° displacement 1 m target	Quick	VOR gain < 0.7 consistent with pathology
**Ross 2016 [37]**	ICS Impulse All canals	10 impulses with 5–10° displacement at ?50°/s 1–2 m target	<30 min	1. Good test–retest reliability 2. Required more impulses to achieve 10 recordable results especially vertical canals 3. Achieving >100°/s velocity difficult—more difficult in vertical canals 4. Limited sensitivity to detect pathology by VOR gain alone due to confounding variables contaminating VOR gain, in particular, vertical canals may be related to maturation of cervical spine 5. Brightly lit room 6. No significant difference in VOR gain across age groups
**Alizadeh 2017 [19]**	Synapsys All canals	10–30° displacement at 160°/s 1 m target	Not given	No difference in VOR gain in gender and age
**Bayram 2017 [21]**	EyeSeeCam Lateral canal only	10 impulses with 5–15° displacement at 100–200°/s 1 m target	Not given	1. Cervical neck stiffness plays a role in VOR gain 2. Factors precluding test were lack of interest, apprehension and fear, and failure of calibration
**Dasgupta 2018 [24]**	ICS Impulse All canals	10 impulses with 10–20° displacement at 100–250°/s 1 m target	10 min	1. Difficult to achieve high velocities in vertical canals 2. Lifting of eyelids 3. Corrective saccades with normal VOR gain 4. Low vertical canal gains due to possible cervical neck contamination 5. Patience and continuous engagement of children and not difficult 6. Should be accompanied by other tests 7. Goggle fitting crucial
**Abdullah 2017 [18]**	ICS Impulse All canals	10–20° displacement at 100–250°/s 1 m target	Quick	1. Instructions are important 2. Lifting of eyelids 3. Easy to perform 4. Additional foam in goggles
**Lehnen 2017 [31]**	EyeSeeCam Lateral canal only	Not given	Less than 10 min	1. Younger children may lack attention to calibrate 2. Covert refixation saccades may occur with normal VOR gain deemed vestibular weakness 3. No age difference in groups
**Wiener-Vacher 2017 [39]**	Synapsys All canals	10–20° displacement at 100–150°/s 1–1.3 m target	Quick	1. Under 4 years, children sit on parents’ lap 2. Instructions important 3. Attractive targets 4. As young as 3 months 5. Difficult to achieve >100°/s in vertical canals, but even then lower peak head velocities do not preclude a similar outcome as that obtained with higher velocities 6. Patience and continuous engagement of children and not difficult 7. Vergence does not play in role across age groups
**Lissner 2019 [32]**	EyeSeeCam Lateral canal only	15 impulses; 10–15° at 150°/s		1. Easy to perform 2. Artifacts generated by moving camera in goggles 3. Blink artifacts
**Emekci 2020 [22]**	ICS Impulse All canals	20 impulses; 10–200° displacement		1. Goggle slippage affects gain 2. Distance from pupil affects gain, ideal between 1 and 1.2 m 3. Position mainly affects younger children
**Retamal 2020 [35]**	EyeSeeCam Lateral canal only	20 impulses		1. Instructions important 2. Having a short attention span 3. Correct placement of head strap and goggles
**Kim 2020 [29]**	ICS Impulse Lateral canal only	10–15 impulses at 10–20° displacement at 100–150°/s		1. Goggle slippage 2. Blink artifacts 3. Normal gain may not indicate normal vestibular function 4. Dependant on operator practice 5. Lack of attention may contaminate VOR gain for tracking
**Abakay 2020 [17]**	ICS Impulse All canals	1 m target		1. Safe and well-tolerated 2. No difference in age groups
**Martens 2022 [33]**	Synapsys All canals	10–20° at 150–250°/s		1. Eminently feasible over 1 year 2. Peak head velocities difficult to achieve in vertical canals 3. Requires considerable experience to interpret results
**Etrugul 2022 [23]**	Synapsys All canals	15–20° at 150–200°/s		1. Interpretation requires experience 2. Gaze errors may contaminate VOR gain 3. Evolution of saccades indicates compensation
**Rodriíguez** **-** **Villalba 2023 [36]**	EyeSeeCam All canals	1 m target		1. VOR gains more homogenous in lateral canals 2. Difficult to achieve velocities in vertical canals 3. Lack of cooperation under 3 years 4. Poor goggle fit 5. Lower vertical canal gains 6. Gains significantly lower in 306 years of age
**Yilmaz 2023 [40]**	ICS Impulse All canals	10–20° displacement		1. Easy to perform 2. Operator variability 3. Goggles’ fit
**Kirbac 2024 [30]**	ICS Impulse All canals	20 impulses; 15–20° displacement at 150–200°/s 1 m target	10 min	Feasible and easy to perform
**Van Hecke 2024 [26]**	ICS Impulse All canals	20 impulses; 10–20° displacement with 120–250°/s 1.5 m target	10–12 min	1. Feasible and quick test 2. Blink artefacts 3. Pulling eyelid for pupil size—wider pupils may affect VOR gain most noticeable to vertical canals—use brightly lit rooms 4. Should be accompanied by other tests 5. Goggle slippage 6. Lack of attention may add noise
**Bachman 2018 [20]**	ICS Impulse All canals	20 impulses; >100°/s	8–17 min	1. More than 50% saccades, magnitude greater than 50% of head amplitude 2. Max time in 4–6 years 3. Eyelid pulled up 4. Attractive targets to engage attention 5. Vertical canals most affected by pupil size 6. Paediatric sized goggles required, and foam used for good fit 7. No difference in age groups in VOR gain
**Zhou 2024 [42]**	ICS Impulse All canals			1. Low vertical canal gains 2. Saccades with normal VOR gain in vertical canals

**Table 2:** VOR gains and study demographics in studies, *n* = 25.

Series	n	Lateral Canal VOR	Vertical Canal VOR	VOR in Pathology
**Hulse 2015 [27]** **EyeSeeCam**	42 (36 + 6); 3–16 years; 32 boys, 23 girls; case–control	1.02 +/− 0.28	Not performed	0.47 +/− 0.3
**Renata 2015 [34]**	28 (19 + 9); 10 boys and 18 girls; 5–18; case–control	Right 0.89 +/− 0.26 Left 0.90 +/− 0.21	RA: 0.96 +/− 0.39 LP 1.03 +/− 0.35 RP: 1.06 +/− 0.33 LA: 1.11 +/− 0.26	RL: 0.75 +/− 0.19 LL: 0.60 +/− 0.33 RA: 1.12 +/− 0.2 LP: 1.23 +/− 0.19 RP: 1.12 +/− 0.26 LA: 0.91 +/− 0.36
**Hamilton 2015 [25]** **ICS Impulse**	33; 3–19 years; 15 boys and 18 girls; only pathology			Range < 0.7 suggested to demonstrate abnormal LSC function of rotatory chair Corrective saccades 100% Vertical canals < 0.7 abnormal
**Khater and Afifi 2016 [28]** **ICS Impulse**	49 (8 + 41); 22 boys and 27 girls; 13–19 years; case–control	Right 1.10 +/− 0.22 Left 1.06 +/− 0.19	RA: 0.96 +/− 0.14 RP: 1.04 +/− 0.11 LA: 0.91 +/− 0.10 LP: 1.03 +/− 0.12	LL: 0.47 +/− 0.21 RL: 0.43 +/− 0.14 LP: 0.38 +/− 0.19 LA: 0.42 +/− 0.24 RP: 0.32 +/− 0.18
**Sommerfleck 2016 [38]** **ICS Impulse**	55; 1–18 years; only pathology	Some normal were found with balance problems	Not performed	0.48 lateral only
**Ross and Helminski 2016 [37]** **ICS Impulse**	28; 4–17 years; only normal	Right Age 4–7 1.04 +/− 0.07 Age 8–12 1.03 +/− 0.05 Age 13–17 1.02 +/− 0.05 Left Age 4–7 1.02 +/− 0.07 Age 8–12 1.01 +/− 0.06 Age 13–17 1.00 +/− 0	RA Age 4–7 1.03 +/− 0.12 Age 8–12 1.08 +/− 0.11 Age 13–17 1.09 +/− 0.11 LA Age 4–7 1.01 +/− 0.10 Age 8–12 1.07 +/− 0.13 Age 13–17 1.07 +/− 0.12 RP Age 4–7 1.04 +/− 0.11 Age 8–12 1.04 +/− 0.11 Age 13–17 1.06 +/− 0.12 LP Age 4–7 1.01 +/− 0.11 Age 8–12 1.06 +/− 0.10 Age 13–17 1.02 +/− 0.07	Not performed
**Alizadeh 2017 [19]** **Synapsys**	60; 6–12 years; 35 boys and 25 girls; only normal	Right lateral 0.99 +/− 0.05 Left lateral 1.00 +/− 0.04	Right anterior 0.98 +/− 0.06 Right posterior 0.94 +/− 0.06 Left anterior 0.98 +/− 0.06 Left posterior 0.94 +/− 0.05	Not performed
**Bayram 2017 [21]** **EyeSeeCam**	100; 6–16 years; 51 boys and 49 girls; only normal	Right Age 6–7 0.9 +/− 0.1 Age 8–12 0.9 +/− 0.1 Age >12 1.00 +/− 0.1 Left Age 6–7 0.9 +/− 0.1 Age 8–12 0.9 +/− 0.1 Age >12 1.00 +/− 0.1	Not performed	Not performed
**Dasgupta 2018 [24]** **ICS Impulse**	100 (39 + 61); 5–16 years; 50 boys and 50 girls; case–control	Left: 0.93 +/− 0.09 Right 0.97 +/− 0.08	LA: 0.65 +/− 0.14 RA: 0.75 +/− 0.14 LP: 0.82 +/− 0.12 RP: 0.69 +/− 0.13	LL: 0.90 +/− 0.18 RL: 0.87 +/− 0.24 LA: 0.6 +/− 0.15 RA: 0.55 +/− 0.24 LP: 0.76 +/− 0.14 RP: 0.53 +/− 0.10
**Abdullah 2017 [18]** **ICS Impulse**	21; 6–15 years; 12 boys and 9 girls; only normal	Right: 0.98 +/− 0.07 Left: 0.94 +/− 0.07	RA: 0.79 +/− 0.14 LP: 0.73 +/− 0.11 LA: 0.84 +/− 0.11 RP: 0.92 +/− 0.12	Not performed
**Lehnen 2017 [31]** **EyeSeeCam**	44; 4–18 years; only normal	4–7 years: 0.96 +/− 0.07	Not performed	Not performed
		8–11 years: 0.95 +/− 0.06		
		12–18 years: 0.94 +/− 0.07		
**Wiener-Vacher and Wiener 2017 [39]** **Synapsys**	274; 1–15 years; only normal	0.966 +/− 0.0789	Anterior: 0.991 +/− 0.0789 Posterior: 0.965 +/− 0.0789	Not performed
**Lissner 2019 [32]** **EyeSeeCam**	33; 13–16 years; 28 boys and 5 girls; only normal	Right: 1.04 +/− 0.10 Left: 1.01 +/− 0.08	Not performed	Not performed
**Emekci 2020** [22] **ICS Impulse**	100; 11–18 years; 50 boys and 50 girls; only normal	Right: 1.00 (+/−0.089); Left: 0.92 (+/−0.104)	RA: 0.84 +/− 0.163 LP: 0.87 +/− 0.122 LA: 0.94 +/− 0.158 RP: 0.86 +/− 0.198	Not performed
**Retamal 2020 [35]** **EyeSeeCam**	39; 5–17 years; only normal	5–10 years: Right: 0.96 +/− 0.21 Left: 1.11 +/− 0.17 11–17 years: Right: 0.90 +/− 0.13 Left: 1.06 +/− 0.18	Not performed	Not performed
**Kim 2020 [29]** **ICS Impulse**	39; 7–18 years; 23 boys and 16 girls; only pathological			<0.8
**Abakay 2020 [17]** **ICS Impulse**	20; 12 -20; only normal	Right 0.96 +/− 0.09 Left 0.85 +/− 0.09	RA: 0.86 +/− 0.06 LA: 0.86 +/− 0.10 RP: 0.92 +/− 0.12 LP: 0.87 +/− 0.05 Ant—0.86 Post—0.89	Not performed
**Martens 2022 [33]** **Synapsys**	133; 5–48 months; 64 boys and 69 girls; only normal	5–12 months: 0.87 +/− 0.08 12–24 months: 0.91 +/− 0.06 24–36 months: 0.92 +/− 0.04 36–48 months: 0.96 +/− 0.04	Anterior: 0.94 +/− 0.06 Posterior: 0.90 +/− 0.06	Not performed
**Ertugrul 2022 [23]** **Synapsys**	40; 1–17 years; only pathological			<0.7
**Rodriíguez-Villalba and Caballero-Borrego 2023 [36]** **EyeSeeCam**	187; 3–16 years; 117 boys and 70 girls; only normal	3–6 years: RH: 0.78 +/− 0.02 LH: 0.76 +/− 0.02 7–10 years: RH: 0.83 +/− 0.04 LH: 0.81 +/− 0.04 11–16 years: RH: 0.82 +/− 0.05 LH: 0.81 +/− 0.04	3–6 years: RA: 0.75 +/− 0.03 LP: 0.72 +/− 0.03 LA: 0.72 +/− 0.04 RP: 0.70 +/− 0.04 7–10 years: RA: 0.78 +/− 0.04 LP: 0.76 +/− 0.04 LA: 0.76 +/− 0.04 RP: 0.74 +/− 0.04 11–16 years: RA: 0.75 +/− 0.08 LP: 0.74 +/− 0.08 LA: 0.72 +/− 0.09 RP: 0.71 +/− 0.08	Not performed
**Yilmaz 2023 [40]** **ICS Impulse**	21 (9 + 12); 5–16 years; 9 boys and 13 girls; case–control	LL: 0.89 +/− 0.1 RL: 0.94 +/− 0.17	LA: 0.84 +/− 0.11 RA: 0.84 +/− 0.13 LP: 0.90 +/− 0.18 RP: 0.86 +/− 0.15	LA: 0.75 +/− 0.07 RA: 0.85 +/− 0.15 LL: 0.77 +/− 0.09 RL: 0.96 +/− 0.14 LP: 0.86 +/− 0.16 RP: 0.85 +/− 0.16
**Kirbac 2024 [30]** **ICS Impulse**	27; 5–14 years; only pathology			Gains < 0.7
**Van Hecke 2024 [26]** **ICS Impulse**	140; 6–13 years; 70 boys and 70 girls; only normal	Right: 1.03 +/− 0.09 Left: 0.97 +/− 0.10 1.0	RA: 0.92 +/− 0.11 LA: 0.92 +/− 0.12 RP: 0.91 +/− 0.15 LP: 0.97 +/− 0.12	Not performed
**Bachman 2018 [20]** **ICS Impulse**	41; 4–12 years; only normal	Right 1.04 +/− 0.09 Left 0.96 +/− 0.09 1.0 Age groups reported 4–6, 7–9, and 10–12 years and averaged	RA 0.9 +/− 0.19 LA 0.8 +/− 0.11 RP 0.83 +/− 0.09 LP 0.91 +/− 0.14	Not performed
**Zhou 2024 [42]** **ICS Impulse**	37 (23 + 14); case–control	Right 1.04 +/− 0.08 Left 0.98 +/− 0.07 1.01 Age groups mentioned 3–7, 8–11, and 13–18 years, not individually analysed but averaged	RA 0.81 +/− 0.15 LA 0.8 +/− 0.15 RP 0.76 +/− 0.08 LP 0.81 +/− 0.14	RL 0.83 +/− 0.3 LL 0.75 +/− 0.27 RA 0.71 +/− 0.23 LA 0.68 +/− 0.26 RP 0.77 +/− 0.24 LP 0.76 +/− 0.24

**Table 3:** Summary of statistical analysis and metanalysis.

Average normal VOR gain across studies for lateral semicircular canals	0.96 +/− 0.07 (range 0.8–1.08)
Average normal VOR gain across studies for anterior semicircular canals	0.89 +/− 0.13 (range 0.74 to 1.03)
Average normal VOR gain across studies for posterior semicircular canals	0.9 +/− 0.12 (range 0.72 to 1.04)
Normal lateral semicircular canal VOR gain comparison between ICS Impulse and EyeSeeCam	*p* > 0.05
Cohen’s d pooled effect sizes for controlled studies	1
Heterogeneity/inconsistencies among controlled studies	I^2^ = 86%

**Table 4:** Recommendations for performing and interpreting vHIT in children—studies with data presented in Table 1 and Table 2 and annotated references in the following table in brackets.

Before Test	During Test and Output
Performed by tester with adequate paediatric experience and practice; skill in executing and interpreting both lateral and vertical canal impulses including artifact recognition (all studies)	10–20° displacement [18,19,20,21,22,23,24,26,27,28,29,30,32,33,34,37,38,39,40,42]
Using attractive targets and play techniques for engaging children [20,24,27,39]	Target at 1–1.5 m [17,18,19,24,26,27,28,30,34,36,37,38,39]
Adequate instruction [18,24,25,26,27,29,32,34,35,39]	100–200° per second head velocity [18,19,20,21,23,24,26,27,28,29,30,32,33,34,37,39]
Brightly lit room [26,34,37]	10–15 head thrusts [21,22,24,25,26,27,28,29,30,32,34,35]
Pulling up eyelids [18,20,24,26]	Average gain as given in Table 3
Using remote controlled camera under 3 years [23,33,39]	Refixation saccade morphology [3,24,28,29,42]

## Data Availability

All data are presented in the article.
